# A Silver‐Induced Absorption Red‐Shifted Dual‐Targeted Nanodiagnosis‐Treatment Agent for NIR‐II Photoacoustic Imaging‐Guided Photothermal and ROS Simultaneously Enhanced Immune Checkpoint Blockade Antitumor Therapy

**DOI:** 10.1002/advs.202306375

**Published:** 2023-12-31

**Authors:** Yulong Bai, Jing Hua, Jingjin Zhao, Shulong Wang, Mengjiao Huang, Yang Wang, Yanni Luo, Shulin Zhao, Hong Liang

**Affiliations:** ^1^ State Key Laboratory for the Chemistry and Molecular Engineering of Medicinal Resources School of Chemistry and Pharmaceutical Science Guangxi Normal University Guilin 541004 China; ^2^ School of Medicine Shanghai Research Institute for Intelligent Autonomous Systems Tongji University Shanghai 200092 China

**Keywords:** hotodynamic therapy, immune checkpoint blockade, nanodiagnosis‐treatment agent, photoacoustic imaging, reactive oxygen species

## Abstract

Tumor metastasis remains a leading factor in the failure of cancer treatments and patient mortality. To address this, a silver‐induced absorption red‐shifted core‐shell nano‐particle is developed, and surface‐modified with triphenylphosphonium bromide (TPP) and hyaluronic acid (HA) to obtain a novel nanodiagnosis‐treatment agent (Ag@CuS‐TPP@HA). This diagnosis‐treatment agent can dual‐targets cancer cells and mitochondria, and exhibits maximal light absorption at 1064 nm, thereby enhancing nesr‐infrared II (NIR‐II) photoacoustic (PA) signal and photothermal effects under 1064 nm laser irradiation. Additionally, the silver in Ag@CuS‐TPP@HA can catalyze the Fenton‐like reactions with H_2_O_2_ in the tumor tissue, yielding reactive oxygen species (ROS). The ROS production, coupled with enhanced photothermal effects, instigates immunogenic cell death (ICD), leading to a substantial release of tumor‐associated antigens (TAAs) and damage‐associated molecular patterns, which have improved the tumor immune suppression microenvironment and boosting immune checkpoint blockade therapy, thus stimulating a systemic antitumor immune response. Hence, Ag@CuS‐TPP@HA, as a cancer diagnostic‐treatment agent, not only accomplishes targeted the NIR‐II PA imaging of tumor tissue and addresses the challenge of accurate diagnosis of deep cancer tissue in vivo, but it also leverages ROS/photothermal therapy to enhance immune checkpoint blockade, thereby eliminating primary tumors and effectively inhibiting distant tumor growth.

## Graphical abstract

A silver‐induced absorption red‐shifted core‐shell nanoparticle is fabricated, and surface‐modified with triphenylphosphonium bromide and hyaluronic acid to obtain a novel nanodiagnosis‐treatment agent (Ag@CuS‐TPP@HA). This diagnosis‐treatment agent not only accomplishes targeted NIR‐II photoacoustic imaging of tumor tissue, but it also leverages ROS/photothermal therapy to enhance immune checkpoint blockade, thereby eliminating primary tumors and effectively inhibiting distant tumor growth.

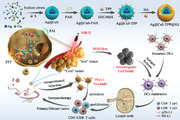

## 1 Introduction

Tumor metastasis is the primary reason behind the failure of cancer treatments and the subsequent mortality in patients. A significant proportion of cancer‐related fatalities can be attributed to the dissemination of primary tumor cells from their origin to distant sites. Among the treatment strategies of cancer, immunotherapy has gained attention for its potential to instigate the body's immune system, revive its functions, and irrevocably eliminate tumor cells. Furthermore, it enhances the patient's systemic immune function to identify, locate, and kill tumor cells, thereby preventing tumor recurrence and metastasis, eradicating primary tumors, and significantly impeding the proliferation of distal tumors. Immune checkpoint blocking (ICB) therapy is an emerging antitumor strategy that has received substantial interest in the biomedical field.^[^ ^1^, ^2^, ^3^, ^4^, ^5^, ^6^ ^]^ This therapy specifically targets immune checkpoints such as cytotoxic T lymphocyte‐associated protein 4 and programmed death receptor 1 (PD‐1) to reactivate T cells and thereby suppress tumor growth. This approach has proven to be effective against a wide range of cancers.^[^ ^7^, ^8^, ^9^, ^10^, ^11^ ^]^ Nevertheless, the therapeutic potential of ICB monoclonal antibody therapy is impeded due to factors such as insufficient T lymphocyte infiltration, the immunosuppressive nature of the tumor microenvironment (TME), and various immune escape mechanisms that may occur in tumor cells.^[^ ^12^, ^13^, ^14^, ^15^ ^]^ To augment the efficacy of ICB therapy, a promising strategy is the induction of immunogenic cell death (ICD), which enhances the antigen presentation of dendritic cells (DCs). This is achieved by releasing tumor‐associated antigens (TAAs) and damage‐associated molecular patterns (DAMPs), leading to further activation, proliferation, and infiltration of T cells in the TME. This process results in the transformation of “cold” tumors into “hot” ones.^[^ ^16^, ^17^, ^18^, ^19^, ^20^, ^21^, ^22^ ^]^ However, relying on a single treatment model is insufficient for the eradication of residual tumor cells and the complete prevention of tumor recurrence. Hence, the design of an integrated system incorporating multiple treatment modalities holds particular appeal for the effective treatment of tumors.

The induction of ICD via chemotherapy is a potential approach to improve tumor cell susceptibility and foster anticancer immunity. However, the application of traditional ICD inducers like chemotherapeutic drugs is often constrained due to their low tumor selectivity and severe adverse effects. Reactive oxygen species (ROS) therapies, such as photodynamic (PDT), sonodynamic (SDT), and chemodynamic (CDT) therapies, are known for their ability to induce ICD.^[^ ^23^, ^24^, ^25^, ^26^, ^27^, ^28^ ^]^ Photothermal therapy (PTT), a modality that transforms light energy into heat energy to eliminate tumor cells, increases the temperature of the tumor microenvironment. Notably, PTT can modulate the tumor microenvironment's immune response by inducing the release of DAMPs via heat shock protein (HSP)‐related reprogramming. These proteins are central to the induction of ICD.^[^ ^29^, ^30^, ^31^, ^32^ ^]^ Furthermore, mitochondrial dysfunction resulting from the targeted disruption of cancer cell mitochondria can expedite DAMP release and subsequently promote ICD.^[^ ^33^ ^]^ Currently, most conventional anticancer drugs interact with signaling pathways situated upstream of mitochondria, congregating on these organelles to instigate cell death. The inhibition of tumor‐specific alterations in mitochondrial metabolism represents a promising treatment avenue. As such, the engineering of mitochondrial targeting nanopharmaceuticals offers an innovative approach to circumvent drug resistance.^[^ ^34^, ^35^ ^]^

Photoacoustic (PA) imaging represents a novel, non‐invasive modality for biomedical imaging.^[^ ^36^, ^37^ ^]^ harnessing the photoacoustic effect to generate 2D or 3D images of biological tissues or materials. Rather than directly detecting light emission, this method relies on the sound waves produced upon absorption of excited light by the intended imaging targets. By amalgamating the advantages of traditional optical and acoustic imaging, PA imaging proffers both the high contrast of optical imaging and the high spatial resolution of ultrasonic imaging. This hybrid imaging approach transcends the inherent limitations of traditional optical imaging by achieving high‐resolution imaging at centimeter depths, thus facilitating high‐resolution optical visualization within deep biological tissues. Moreover, the utilization of PA imaging eliminates the hazards associated with ionizing radiation and invasive procedures. As such, it does not perturb normal tissues, thereby minimizing risk during the imaging process. Accordingly, PA imaging has been recognized as a pioneering biomedical imaging technology with vast potential for clinical visual diagnoses and treatment monitoring. Notably, PA imaging in the near‐infrared zone II (NIR‐II, 1000–1700 nm) presents a unique advantage in its ability to more precisely locate tumor tissue and guide cancer treatment. This superior accuracy is attributed to the reduced interference from biological tissue background signals, the enhanced imaging resolution, and the increased penetration depth within biological tissues.^[^ ^38^, ^39^, ^40^, ^41^, ^42^, ^43^ ^]^

**Scheme 1:**
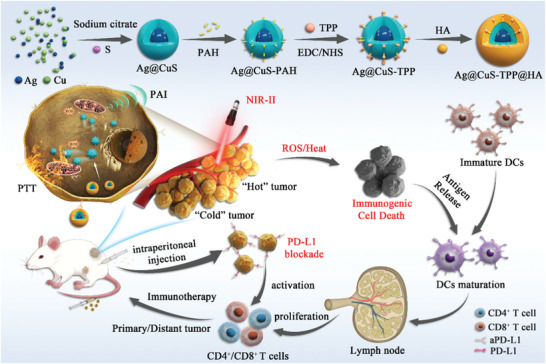
The synthesis process of Ag@CuS‐TPP@HA nanoparticles and the principle of enhanced antitumor therapy. The diagram illustrates how ROS/PTT induce ICD, increase cytotoxic T lymphocyte infiltration at the tumor site, induce a systemic antitumor immune response, and integrate with ICB to effectively inhibit the growth of distal tumors.

In this study, we developed novel cancer cells and mitochondria dual‐targeted nanodiagnosis‐treatment agent, Ag@CuS‐TPP@HA, with targeted capabilities toward cancer cells and their mitochondria. The initial stage involved preparing an Ag@CuS core‐shell nano‐enzyme, followed by surface modification using triphenylphosphorus bromide (TPP) and hyaluronic acid (HA), resulting in a multifunctional nanoparticle. The unique design of this nanoparticle allows the simultaneous triggering of its CDT/PTT by the TME and NIR light irradiation, initiation of ICD in tumor cells, and coordination with ICB antibodies to activate a systemic antitumor immune response. In the complex, Ag@CuS‐TPP@HA, the CuS‐coating over Ag extends its absorption spectrum from the NIR I to the NIR II region, and enhancing photothermal properties. The hyaluronic acid present on the surface enables the nano‐diagnostic agent to target the overexpressed CD44 receptors on cancer cells, leading to a high concentration at the tumor site. Additionally, the Ag in Ag@CuS‐TPP@HA can catalyze the Fenton‐like reactions with H_2_O_2_ in the tumor tissue, yielding ROS. The ROS production, coupled with enhanced photothermal effects, instigates ICD, leading to a substantial release of TAAs and DAMPs. The targeted NIR‐II PA imaging and CDT/PTT of the tumor can be achieved under the irradiation of an NIR‐II laser. In the presence of hyaluronidase (HAase) within the TME, the hyaluronic acid is digested, causing charge reversal on the nano‐diagnostic agent's surface. This process further targets the mitochondria within tumor cells, causing mitochondrial dysfunction under NIR‐II light, thereby inducing cancer cell death and improving CDT/PTT therapeutic effects. This also results in the concurrent triggering of ICD and the subsequent release of TAAs and DAMPs, promoting the maturation of DCs and the infiltration of CTLs into the tumor, thereby improving the tumor immunosuppressive microenvironment. The synergy of ICD and ICB induced by CDT/PTT effectively reverses the tumor's immunosuppressive microenvironment and stimulates a robust immune response. This comprehensive approach leads to the complete elimination of the primary tumor and significantly inhibits distal tumor growth (**Scheme** **1**).

## 2 Results and Discussion

### 2.1 Preparation and Characterization of Ag@CuS‐TPP@HA Nanoparticles

**Figure 1:**
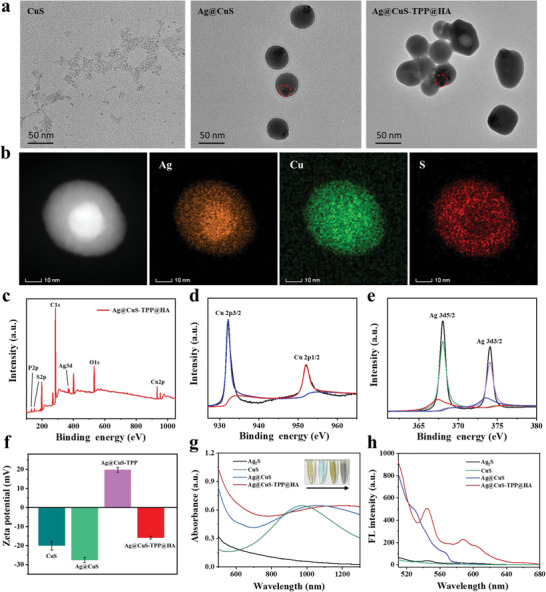
Characterization of Ag@CuS‐TPP@HA. a) TEM images of CuS, Ag@Cu, and Ag@CuS‐TPP@HA nanoparticles. b) EDS element mapping images of Ag@CuS core‐shell nanoparticles. c XPS maps of Ag@CuS‐TPP@HA. d,e) XPS spectra of bivalent copper and monovalent silver in Ag@CuS‐TPP@HA and its structure. f) Zeta potential maps of CuS, Ag@CuS, Ag@CuS‐TPP, and Ag@CuS‐TPP@HA nanoparticles. g) Absorption spectra and h) fluorescence spectra (λ_ex_ = 405 nm) of Ag_2_S, CuS, Ag@CuS, and Ag@CuS‐TPP@HA nanoparticles. Error bars represent the standard deviation of three independent measurements.

Ag@CuS‐TPP@HA nanoparticles were synthesized via a two‐stage procedure. Initially, water‐soluble Ag@CuS core‐shell nanoparticles were produced using CuCl_2_, sodium citrate, Na_2_S, and AgNO_3_ as precursors. Subsequently, these obtained nanoparticles underwent a reaction with polyallylamine hydrochloride (PAH), (4‐carboxybutyl) triphenylphosphorus bromide (TPP), and HA to yield Ag@CuS‐TPP@HA nanoparticles. The formation of these nanoparticles was facilitated by electrostatic interactions and amide bond formation. The morphological characteristics and particle sizes of CuS, Ag@CuS, and Ag@CuS‐TPP@HA nanoparticles were explored using transmission electron microscopy (TEM). **Figure** **1**a depicts the resultant images. The TEM examination reveals CuS as quasi‐spherical nanoparticles with effective dispersion, exhibiting an average particle size of approximately 4 nm. The Ag@CuS nanoparticles displayed a spherical core‐shell structure with an average particle size of roughly 44.5 nm, which is approximately 11‐fold larger than that of CuS nanoparticles. Following surface modification of Ag@CuS with TPP and HA, the particle size was further amplified to 50.4 nm. Energy dispersive X‐ray spectroscopy (EDS) provided evidence of the core‐shell structure of Ag@CuS nanoparticles, as demonstrated by element mapping images (Figure 1b). X‐ray photoelectron spectroscopy (XPS) was utilized to characterize the elemental composition of CuS, Ag@CuS, and Ag@CuS‐TPP@HA nanoparticles. As depicted in Figure S1 (Supporting Information), CuS nanoparticles comprised C, O, S, and Cu; Ag@CuS nanoparticles consisted of C, O, S, Ag, and Cu; while Ag@CuS‐TPP@HA nanoparticles contained C, O, S, P, Ag, and Cu (Figure 1c). These results corroborate the successful synthesis of Ag@CuS‐TPP@HA nanoparticles. Additional analysis of the Cu and Ag chemical states revealed that the central positions of the Cu2p1/2 and Cu2p3/2 peaks in Ag@CuS‐TPP@HA were located at 952.0 and 932.1 eV (Figure 1d), respectively. Correspondingly, the central positions of the Ag3d3/2 and Ag3d5/2 peaks were at 374.05 and 368.04 eV (Figure 1e), respectively. The peak positions can be ascribed to the characteristic peaks of Cu (II) and elemental Ag in the Ag@CuS‐TPP@HA composite, further confirming the nano‐Ag core formation and substantiating the core‐shell structure of the Ag@CuS‐TPP@HA nanoparticles. Throughout the Ag@CuS‐TPP@HA synthesis process, a double reversal of the Zeta potential of nanoparticles was observed. Initially, the potential shifted from −27.5 eV of Ag@CuS to 19.7 eV of Ag@CuS‐TPP, before reversing to −15.8 eV of Ag@CuS‐TPP@HA (Figure 1f). These charge reversal phenomena can be attributed to the surface charge alterations in nanoparticles, which were induced by the positive charge of TPP on the Ag@CuS surface and the negative charge of HA.

To explore the optical attributes of Ag@CuS‐TPP@HA, we conducted measurements of both absorption and fluorescence spectra (λex = 405 nm) for Ag2S, CuS, Ag@CuS, and Ag@CuS‐TPP@HA nanoparticles. The data revealed that the optical absorption peak for CuS nanoparticles is situated at approximately 950 nm, whereas Ag@CuS core‐shell nanoparticles doped with Ag have a red‐shifted absorption peak at about 1100 nm. This shift can likely be attributed to the Ag doping, which can enhance the carrier density and stimulate electron transfer, resulting in an effective reduction of the bandgap. Consequently, this leads to a red shift in the absorption spectra of Ag@CuS and induces a change in solution color from light brown to dark brown. Upon modification with TPP and HA, the solution color of Ag@CuS‐TPP@HA is altered to a deep brown, bordering on black (Figure 1g). Contrarily, under the excitation of light with a 405 nm wavelength, neither Ag_2_S nor CuS nanoparticles solutions exhibit fluorescence emission. However, the Ag@CuS nanoparticles solution has a pronounced fluorescence emission in the 520–580 nm wavelength range.^[^ ^44^ ^]^ Post surface modification with TPP and HA, the fluorescence emission spectrum of Ag@CuS‐TPP@HA solution undergoes an additional red shift (Figure 1h).

### 2.2 Chemical Kinetics and Photothermal/Photoacoustic Properties of Ag@CuS‐TPP@HA

**Figure 2:**
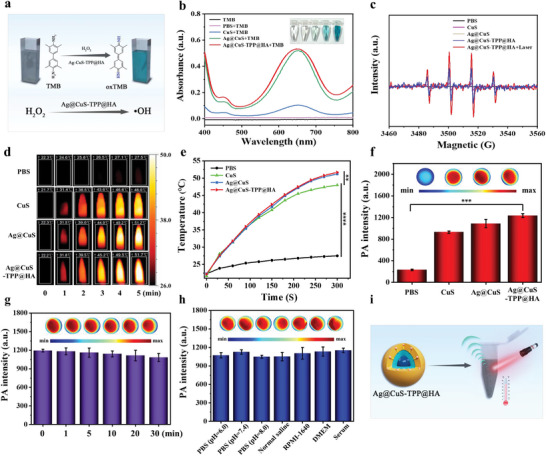
The chemodynamic and photothermal/PA properties of Ag@CuS‐TPP@HA. a) Schematic of ∙OH generation via the TMB method. b) Absorption spectra of TMB in the presence of different species. c) ∙OH production under various conditions assessed by electron paramagnetic resonance (EPR) spectroscopy. d) Thermal imaging of different substances under 1064 nm laser irradiation. e) Temperature increase curves of different substances under 1064 nm laser irradiation. f) PA imaging and PA intensity of different substances under 1064 nm laser irradiation (Material concentration: 400 µg mL^−1^). g) PA signal stability of Ag@CuS‐TPP@HA solution under 1064 nm laser irradiation. h) PA signal stability in various physiological media. i) PA imaging/photothermal heating diagram in vitro. Laser power is 1.5 W cm^−2^. Error bars represent the standard deviation of three independent measurements. Student's *t*‐test is used for statistical analysis, ^****^ *p* < 0.0001, ^**^ *p* < 0.01.

Capitalizing on the distinct core‐shell structure and the near‐infrared bi‐zone optical absorption traits of Ag@CuS‐TPP@HA, its capacity for ROS production and its photothermal/PA attributes were ascertained. First, the capacity for ROS production was ascertained utilizing 3,3′,5,5′‐tetramethylbenzidine (TMB) absorption spectroscopy in conjunction with electron paramagnetic resonance (EPR) spectroscopy. These investigations revealed that Ag@CuS‐TPP@HA can induce a redox reaction of H_2_O_2_, producing ∙OH which is capable of transforming the colorless TMB into blue oxTMB, as manifested by a notable absorption peak at 652 nm (**Figure** **2**a). When Ag@CuS and Ag@CuS‐TPP@HA were reacted with H_2_O_2_ and TMB, there was a significant increase in the light absorption intensity of the solution (Figure 2b) compared to CuS nanoparticles alone. This indicates the presence of nano‐enzyme catalytic activity in both Ag@CuS and Ag@CuS‐TPP@HA, leading to ∙OH production. EPR spectral analysis results further substantiate that Ag@CuS‐TPP@HA can generate an elevated amount of ∙OH under the influence of 1064 nm laser irradiation. This phenomenon can be credited to the photothermal effect of Ag@CuS‐TPP@HA, which expedites the ROS reaction rate (Figure 2c). We further discussed whether Ag@CuS‐TPP@HA can generate singlet oxygen for photodynamic therapy. We took 0.0027 g of 1,3‐diphenylisobenzofuran (DPBF) and dissolved it in 2 mL of absolute ethanol. The Ag@CuS‐TPP@HA solution and DPBF (0.1 mm) were mixed in phosphate buffer, and irradiated with a 1064 nm (1.0 W cm^−2^) laser, and the UV–vis absorption curve of the DPBF solution was recorded. Experimental results found that the UV absorption peak at 420 nm of the Ag@CuS‐TPP@HA solution changed slightly after irradiation for 5 min (Figure S2, Supporting Information), indicating that the Ag@CuS‐TPP@HA nanocomposite produced less singlet oxygen, so The main type of active oxygen produced in the system is **·**OH. Collectively, these findings demonstrate that Ag@CuS‐TPP@HA can instigate a Fenton‐like reaction involving Ag and H_2_O_2_, resulting in ∙OH production. Concurrently, the photothermal impact of ROS and Ag@CuS‐TPP@HA can trigger ICD.

To assess the potential of Ag@CuS‐TPP@HA nanoparticles as a near‐infrared II (NIR‐II) light‐activated PTT agent, we conducted an in vitro experiment monitoring the temperature changes of the nanocomposite solution using an infrared thermal imager under NIR‐II light irradiation (Figure 2d,i). Upon laser irradiation at 1064 nm (1.0 W cm^−2^), the temperature of the Ag@CuS‐TPP@HA solution (400 µg mL^−1^) progressively rose over time, reaching a peak of 51.7 °C within 5 min (Figure 2e). We further investigated the PA properties of various substances under NIR‐II light. Figure 2f presents the PA images of CuS, Ag@CuS, and Ag@CuS‐TPP@HA solutions (400 µg mL^−1^) under 1064 nm laser irradiation. Notably, the PA intensities of these solutions increased sequentially, indicating their robust PA characteristics under NIR‐II. The Ag@CuS‐TPP@HA solution exhibited the strongest PA response, correlating with its superior photoabsorption properties in the NIR region (Figure 1g). Additionally, we explored the stability of the PA signal for the Ag@CuS‐TPP@HA solution under 1064 nm laser irradiation and its stability across various physiological media. Our data revealed that the PA signal intensity remained largely unaltered over 30 min when irradiated at 1064 nm (1.0 W cm^−2^) (Figure 2g). A similar stability pattern was observed across different solvents (PBS, saline, RPMI‐1640, DMEM, and fetal bovine serum) (Figure 2h). Collectively, our findings suggest that Ag@CuS‐TPP@HA exhibits robust chemical kinetics and photothermal/PA properties. Therefore, it holds promise as an efficient nano‐diagnostic agent for tumor treatment guided by NIR‐II PA imaging.

### 2.3 Intracellular ROS Production and Immunogenic Cell Death (ICD)

**Figure 3:**
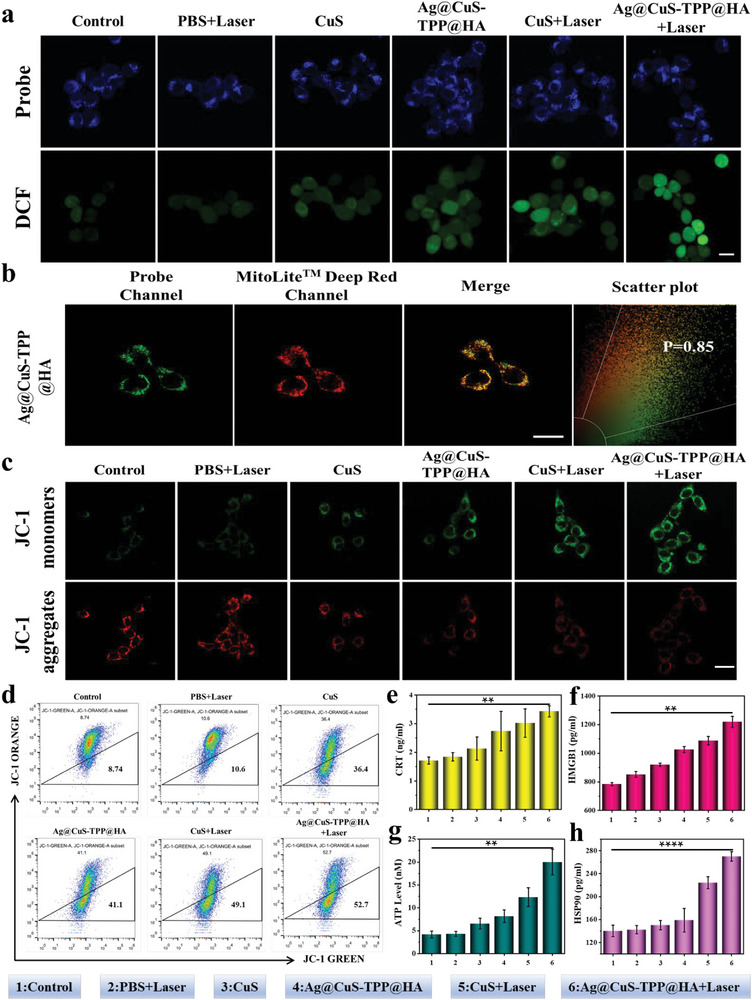
Intracellular responses to various treatments. a) Intracellular ROS production under different treatment conditions (blue channel: λ_ex_ = 405 nm, λ_em_ = 415–650 nm; green channel: λ_ex_ = 488 nm, λ_em_ = 498–650 nm). Scale bar: 10 µm. b) Mitochondrial co‐localization cellular imaging of Ag@CuS‐TPP@HA (green channel: λ_ex_ = 405 nm, λ_em_ = 415–650 nm; red channel: λ_ex_ = 633 nm, λ_em_ = 643–800 nm). Scale bar: 20 µm. c) Cytofluorescence imaging of intracellular mitochondrial membrane potential under different treatment conditions (green channel: λ_ex_ = 488 nm, λ_em_ = 498–550 nm; red channel: λ_ex_ = 552 nm, λ_em_ = 562–650 nm). Scale bar: 10 µm. d) Flow cytometry analysis results. e–h) CRT, HMGB1, ATP, and HSP90 release levels post various treatments (*n* = 3). Error bars represent the standard deviation of three independent measurements, analyzed using the Student's *t*‐test, ^****^ *p* < 0.0001, ^**^ *p* < 0.01.

To elucidate the ROS generation capacity of Ag@CuS‐TPP@HA, we gauged the intracellular ∙OH production using two‐photon laser confocal imaging. This was achieved by employing 2,7‐dichlorodihydrofluorescein diacetate (DCFH‐DA) as the probe. As **Figure** **3**a illustrates, among the six groups under investigation, the control group and PBS+Laser group exhibited minimal green fluorescence. In contrast, both the CuS group and Ag@CuS‐TPP@HA group demonstrated a significant increase in green fluorescence, indicative of heightened ROS levels. However, the Ag@CuS‐TPP@HA+Laser group displayed the most pronounced green fluorescence, suggesting maximal ROS levels within this group. Complementing these observations, flow cytometric analysis corroborated the results of the confocal imaging, as shown in Figure S3 (Supporting Information). These combined findings underline the superior ROS production ability of Ag@CuS‐TPP@HA.

The dual targeting potential of Ag@CuS‐TPP@HA toward tumor cells and their mitochondria was confirmed by a series of experimental observations. To illustrate this, 4T1 cells were exposed to both Ag@CuS and Ag@CuS‐TPP@HA for two, four, and six‐hour intervals, and resultant fluorescence signal intensities were measured (Figure S4, Supporting Information). The Ag@CuS‐TPP@HA group exhibited a time‐dependent increase in fluorescence intensity, peaking at the four‐hour mark. In contrast, the Ag@CuS group showed negligible fluorescence, barely detectable even at 2 h. This suggests that the HA and CD44 receptor interaction on tumor cell surfaces facilitates the uptake of Ag@CuS‐TPP@HA, confirming its active tumor‐targeting ability.^[^ ^42^ ^]^ Further investigations were carried out to explore the mitochondrial targeting potential of Ag@CuS‐TPP@HA. 4T1 cells were co‐incubated with Ag@CuS‐TPP@HA and MitoLite Deep Red FX660, a mitochondrial targeting probe (Figure 3b). The observed significant fluorescence overlap between Ag@CuS‐TPP@HA and MitoLite Deep Red FX660, alongside a Pearson correlation coefficient of 0.85, compared to a mere 0.62 for Ag@CuS (Figure S5, Supporting Information), can be attributed to the substantial positive charge provided by the TPP modifications in Ag@CuS‐TPP@HA.^[^ ^43^ ^]^ Ag@CuS‐TPP@HA is shown to produce numerous highly toxic ROS within the mitochondria, leading to mitochondrial dysfunction. This was further substantiated by JC‐1 staining experiments which monitor changes in mitochondrial membrane potential (Figure 3c). Compared to the control group's bright red fluorescence indicative of healthy mitochondria, the Ag@CuS‐TPP@HA group exhibited a significantly enhanced green fluorescence, suggesting mitochondrial damage. Following laser irradiation, the Ag@CuS‐TPP@HA+Laser group's green fluorescence peaked with a corresponding decrease in red fluorescence. This indicates that ROS/PTT helps to diminish MMP, a conclusion that aligns with the flow cytometry (FCM) analysis results (Figure 3d). Thus, it is evident that Ag@CuS‐TPP@HA can generate substantial ROS within the mitochondria, contributing to oxidative mitochondrial damage.

We next investigated the capability of CuS nanoparticles and the combination of Ag@CuS‐TPP@HA and NIR‐II light to trigger ICD in tumor cells. ICD induction in tumor cells leads to the production of a sequence of signal molecules known as damage‐associated molecular patterns (DAMPs). DAMPs include cell surface exposed calreticulin (CRT), high mobility group protein 1 (HMGB1) secreted by tumor cells, adenosine triphosphate (ATP) molecules released by tumor cells, heat shock protein (HSP90), among others. The extent of CRT exposure, HMGB1 release, ATP secretion, and HSP90 overexpression were quantitatively analyzed using the enzyme‐linked immunosorbent assay (ELISA).^[^ ^19^, ^21^, ^23^ ^]^

CRT emerges on the cell membrane surface acting as an “eat‐me” signal, supplying ample antigenic substances to foster DC maturation and functionality. As demonstrated in Figure 3e, CRT expression exhibited a 1.25 and 1.60‐fold increase in the CuS nanoparticles and Ag@CuS‐TPP@HA groups, respectively. When coupled with laser application, the increase in CRT expression was amplified to 1.76 and 2.01‐fold in the CuS+Laser and Ag@CuS‐TPP@HA+Laser groups, respectively, reflecting apoptosis levels. HMGB1, released from the nucleus during mechanical injury, initiates the corresponding signal pathway, thus stimulating an immune response. Compared to the control group, HMGB1 release in the CuS nanoparticles and Ag@CuS‐TPP@HA groups increased by 17.3% and 30.8%, respectively (Figure 3f). The increase was more pronounced, reaching 38.7% and 55.5%, respectively, in the CuS+Laser and Ag@CuS‐TPP@HA+Laser groups, aligning with the extent of cellular damage. Apoptosis of tumor cells triggers the extracellular release of ATP, delivering a “find‐me” signal. This release facilitates DC cell maturation, and immune cell infiltration, and subsequently stimulates specific antitumor immune responses.^[^ ^42^ ^]^ Our data revealed that intracellular ATP levels surged 1.56 and 1.96‐fold in the CuS nanoparticles and Ag@CuS‐TPP@HA groups, respectively. After exposure to light, the increase rose to 2.95 and 4.78‐fold, respectively (Figure 3g). HSP90, a cellular stress protein, can migrate from the nucleus to the cytoplasm under high thermal or other stress conditions before being discharged into the extracellular space. Compared to the control group (Figure 3h), the HSP90 level in the CuS nanoparticles and Ag@CuS‐TPP@HA groups only slightly increased. However, in the CuS+Laser and Ag@CuS‐TPP@HA+Laser groups, the increase was significant, rising 1.60 and 1.92‐fold respectively, implying a dominant role of the thermal effect in HSP90 expression. This further corroborates that Ag@CuS‐TPP@HA can efficiently stimulate the generation of ROS in mitochondria, inducing ICD and prompting an antitumor immune response.

### 2.4 In Vitro Therapy, PA/Photothermal Imaging, and Evaluation of Biological Distribution and Metabolism In Vivo

**Figure 4:**
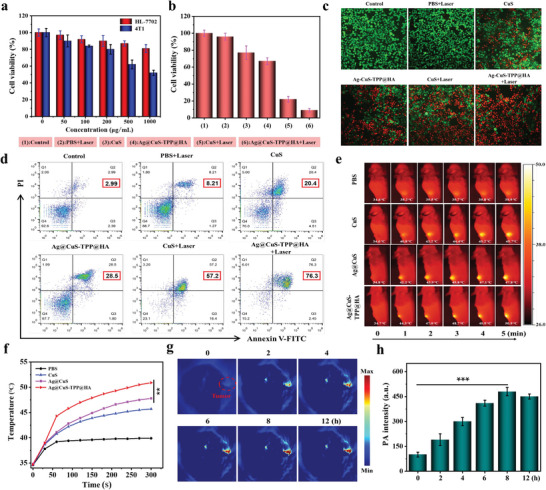
Treatment effects on cell survival, apoptosis, and in vivo thermal imaging. a) Survival rate of cells exposed to varying concentrations of Ag@CuS‐TPP@HA for 24 h. b) Survival rate of 4T1 cells exposed to different substances for 24 h. c) Viability/death staining of cells. d) Apoptosis rates assessed by flow cytometry. e) In vivo photothermal imaging of tumor tissue in mice post‐tail vein injection of PBS, CuS, Ag@CuS, and Ag@CuS‐TPP@HA (1 mg mL^−1^). f) Tumor tissue temperature changes over time with laser irradiation following tail vein injection of PBS, CuS, Ag@CuS, and Ag@CuS‐TPP@HA. g) PA imaging of tumor tissue in mice after tail vein injection of Ag@CuS‐TPP@HA (1 mg mL^−1^). h) Changes in PA signal intensity of tumor tissue in mice post‐tail vein injection of Ag@CuS‐TPP@HA. Error bars denote the standard deviation of three independent measurements. Student's *t*‐test used for statistical analysis, ^***^ *p* < 0.001, ^**^ *p* < 0.01.

Recognizing the promising ROS production and photothermal attributes of Ag@CuS‐TPP@HA, we proceeded to investigate its impact on cancer cells both in vitro and in vivo, specifically assessing its potential in photoacoustic and photothermal imaging applications. The cytotoxicity of Ag@CuS‐TPP@HA toward 4T1 cells (mouse breast cancer cells) and HL‐7702 cells (normal human liver cells) was first ascertained through the MTT assay. Cells were exposed to a range of concentrations (0, 50, 100, 200, 500, 1000 µg mL^−1^) of Ag@CuS‐TPP@HA over a 24 h incubation period, after which the survival rates of both cell types were separately determined. Findings indicated that, after a 24 h incubation with a high concentration (1000 µg mL^−1^) of Ag@CuS‐TPP@HA, the survival rate of HL‐7702 cells remained above 80%, while that of the 4T1 cells dropped to 52% (**Figure** **4**a). This substantiated the nanoparticle's high biocompatibility with normal cells and its cytotoxicity toward cancer cells. The survival rates of 4T1 cells under different treatment conditions were evaluated by the MTT method. As can be seen from the Figure 4b and Figure S6a (Supporting Information), the survival rates of the Au@CuS‐TPP group and the Ag@CuS@HA group cells are similar, but both are higher than the 65% in the Au@CuS‐TPP@HA group (Figure 4b), which confirms the superior therapeutic effect of the dual targeting of the Au@CuS‐TPP@HA group. However, following 5 min of laser irradiation, the survival rate plummeted to below 25%, most markedly in the Ag@CuS‐TPP@HA+Laser group. This affirms the heightened efficacy of Ag@CuS‐TPP@HA under laser irradiation in treating cancer cells. Finally, live/dead cell staining methods were employed to visually assess apoptosis among 4T1 cells under different treatment conditions. Results showed that the number of apoptotic cells in Au@CuS‐TPP group and Ag@CuS@HA group was significantly less than that in Ag@CuS‐TPP@HA group (Figure S6b, Supporting Information), which further confirmed the superiority of double targeting therapy. We found that Ag@CuS‐TPP@HA+Laser group cells almost completely apoptosis (Figure 4c), which is consistent with the results of flow cytometry apoptosis detection (Figure 4d).

To assess the photothermal and PA imaging potential of Ag@CuS‐TPP@HA in a living system, we introduced the Ag@CuS‐TPP@HA solution into mice via tail vein injection. Subsequent in vivo NIR‐II (1064 nm) PA imaging was then performed over a 12 h monitoring period (Figure 4g). It was observed that the photoacoustic signal intensity peaked at the tumor site 8 h post‐injection, suggesting maximum accumulation of Ag@CuS‐TPP@HA at the tumor location (Figure 4h). Thus, the optimal time for both monitoring and treatment was identified as 8 h following the administration of the Ag@CuS‐TPP@HA solution. Following this, in vivo photothermal imaging of the tumor tissue was conducted at this optimal monitoring time. As demonstrated in Figure 4e, the tumor temperature increased in response to extended laser (1.0 W cm^−2^) irradiation across the three groups (CuS nanoparticles, Ag@CuS, and Ag@CuS‐TPP@HA). Upon 5 min of laser irradiation, the respective tumor temperatures rose to 45.5, 47.8, and 50.9 °C (Figure 4f). The Ag@CuS‐TPP@HA group recorded the highest temperature, attributable to the targeted tumor interaction via the cell surface differentiation cluster 44 (CD44) receptor by the HA present on the Ag@CuS‐TPP@HA surface, thereby enhancing tumor cell uptake of Ag@CuS‐TPP@HA.^[^ ^45^ ^]^ Consequently, Ag@CuS‐TPP@HA demonstrated promising PA/photothermal imaging performance in vivo, providing a basis for an accurate, integrated diagnostic and therapeutic approach.

To discern the in vivo biodistribution and metabolism of Ag@CuS‐TPP@HA, inductively coupled plasma atomic emission spectrometry (ICP‐OES) was utilized (Figure S7, Supporting Information). Initial biodistribution data indicated that Ag@CuS‐TPP@HA predominantly accumulated in the liver and lung tissues within 2 h of injection. However, after 24 h, accumulation in these organs decreased significantly, while remaining relatively unchanged in the tumor tissue. This suggests that the majority of Ag@CuS‐TPP@HA nanoparticles distributed across various biological organs (heart, liver, spleen, lung, and kidney) could be metabolized following a 24‐hour circulation period in vivo but remained stable within the tumor tissues. A large amount of Cu and Ag components were detected to be excreted from the tumor‐bearing mice through urine and feces (Figure S8, Supporting Information), indicating that Ag@CuS‐TPP@HA can be safely used for photoacoustic imaging and treatment of tumor tissues in vivo. This observation supports the safe application of Ag@CuS‐TPP@HA for in vivo PA imaging and tumor treatment.

### 2.5 ROS/PTT‐Enhanced Immune Checkpoint Blockade in Antitumor Therapy

**Figure 5:**
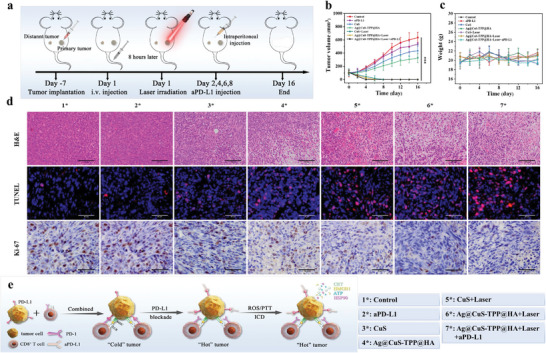
Effects of various treatments on tumor growth, body weight, and histological markers in BALB/c tumor‐bearing mice. a) Schematic of the treatment process for primary and distal tumors. b) Changes in primary tumor volume over 16 days of treatment across different groups. c) Changes in body weight of tumor‐bearing mice across different treatment groups over the 16‐day treatment period. d) Post two rounds of treatment, tumor tissues of mice from different groups were stained for H&E, TUNEL immunofluorescence, and Ki‐67 immunohistochemical analysis. e) Schematic of ROS/PTT‐enhanced immune checkpoint blockade antitumor therapy. Error bars represent the standard deviation of five independent measurements. Statistical analysis was performed using the Student's *t*‐test, ^***^ *p* < 0.001.

Given the high cytotoxicity of Ag@CuS‐TPP@HA toward tumor cells, we expanded our study to investigate its in vivo antitumor effects and its potential to enhance immune checkpoint blockade therapy (**Figure** **5**a). 4T1 cells were subcutaneously injected into both sides of the mice's back to simulate primary and distal tumor disease. We then treated these tumor‐bearing mice with various therapeutic agents, tracking the changes in primary tumor volume over time (Figure 5b). The results indicated only a slight inhibition of primary tumor growth in the aPD‐L1 treatment group. In contrast, the Ag@CuS‐TPP@HA and CuS treatment groups demonstrated significant primary tumor growth inhibition. Notably, the Ag@CuS‐TPP@HA+Laser+aPD‐L1, Ag@CuS‐TPP@HA+Laser, and CuS+Laser treatment groups exhibited complete primary tumor ablation by the fourth, sixth, and eighth day of treatment, respectively. Among these, the Ag@CuS‐TPP@HA+Laser+aPD‐L1 treatment proved the most efficacious. Throughout the treatment period, the body weight of the BALB/c tumor‐bearing mice remained relatively stable (Figure 5c). To further elucidate the tumor damage caused by the different treatments, we performed hematoxylin‐eosin (HE), terminal deoxynucleotidyl transferase‐mediated dUTP nick‐end labeling (TUNEL), and Ki‐67 cell proliferation index imaging on tumor tissues from each treatment group (Figure 5d). We observed markedly higher nuclear pyknosis, deep staining, and necrosis in the Ag@CuS‐TPP@HA+Laser+aPD‐L1 group compared to the control group and other treatment groups, findings that corroborate the results from TUNEL detection. Ki‐67 detection further indicated an almost complete halt in tumor proliferation in the Ag@CuS‐TPP@HA+Laser+aPD‐L1 group.

**Figure 6:**
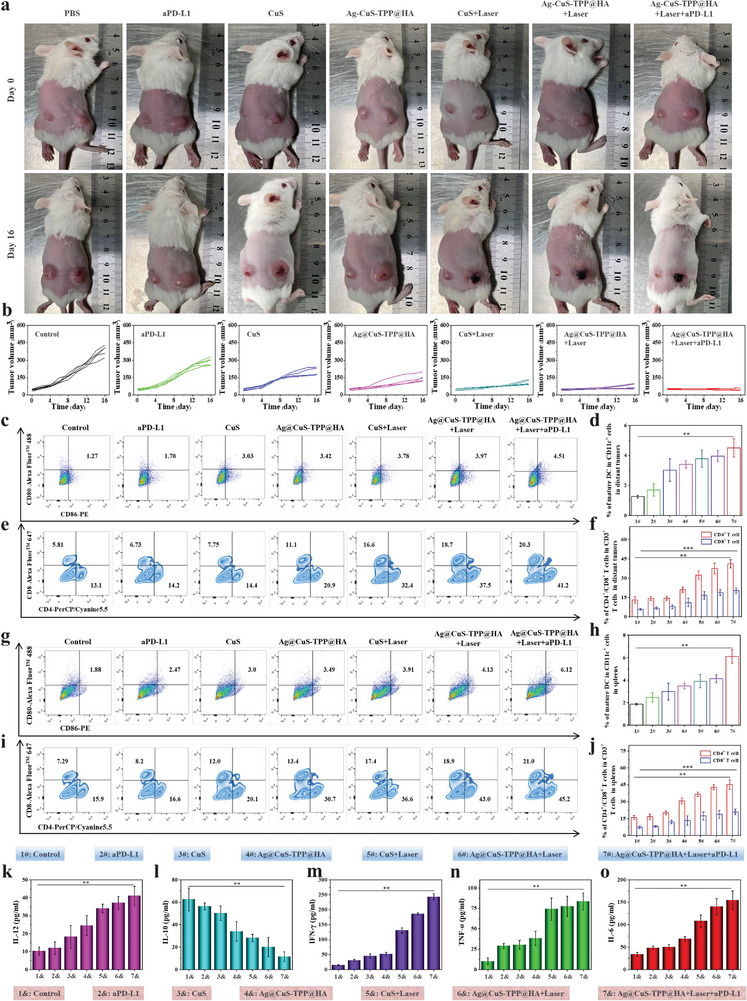
Impact of different therapeutic agents on distal tumor volume, dendritic cell maturation, and T lymphocyte infiltration. a) Digital photos of BALB/c tumor‐bearing mice before and 16 days after treatment with different therapeutic agents. b) Changes in distal tumor volume during treatment in tumor‐bearing mice (*n* = 5). c,d) Flow cytometry analysis of tumor tissue cell suspensions collected on day 8 post‐treatment, stained with CD11c, CD80, and CD86. e,f) Assessment of dendritic cell maturation rate and infiltration of CD4^+^ and CD8^+^ T lymphocytes (with CD3^+^ T cells as gating). Data expressed as average (*n* = 3). g,h) Flow cytometry analysis of spleen tissue cell suspension collected on day 8 post‐treatment, stained with CD11c, CD80, and CD86. i,j)Evaluation of dendritic cell maturation rate and infiltration of CD4^+^ and CD8^+^ T lymphocytes (with CD3^+^ T cells as gating). Data expressed as average (*n* = 3). k–o) ELISA‐based measurement of serum levels of IL‐12, IL‐10, IFN‐ γ, TNF‐ α, and IL‐6 in mice from each treatment group (*n* = 3). Error bars represent the standard deviation of three independent measurements. Statistical analysis was performed using the Student's *t*‐test, ^***^ *p* < 0.001, ^**^ *p* < 0.01.

In order to determine whether ROS/PTT‐enhanced immune checkpoint blockade antitumor therapy strategy can stimulate the immune response to distal tumors, we deployed both a singular therapeutic agent and a combination therapy for the treatment of 4T1 tumor‐bearing BALB/c mice (Figure 5e). The treatment's effect on five tumor‐bearing BALB/c mice is documented in **Figure** **6**b. From the illustrated data, it becomes evident that singular therapeutic agents (aPD‐L1, CuS, Ag@‐CuS‐TPP@HA) show a limited effect on distal tumors. However, when employing combined therapies (CuS+Laser, Ag@CuS‐TPP@HA+Laser, Ag@CuS‐TPP@HA+Laser+aPD‐L1), there is a significant slowdown in distal tumor growth within tumor‐bearing mice. Notably, the Ag@CuS‐TPP@HA+Laser+aPD‐L1 combination therapy nearly halts distal tumor growth entirely. Supporting our observations, the therapeutic impact of each treatment group on the mice can be corroborated through the comparison of digital images captured before and after treatment (Figure 6a). These findings demonstrate that the combination of ROS/PTT and aPD‐L1 therapy is crucial in enhancing the immune system's response, thereby inhibiting distal tumor growth.

To elucidate the antitumor mechanism of combined ROS/PTT and aPD‐L1 treatment, we conducted further investigations into the immune cells present in both tumor and spleen tissues, as well as cytokines present in the serum of tumor‐bearing mice. The proportion of tumor‐infiltrating mature DCs was assessed using flow cytometry (Figure 6c). Notably, the percentage of mature DCs (characterized by CD11c^+^ CD80^+^ CD86^+^ markers) infiltrating the tumors was significantly higher in the Ag@CuS‐TPP@HA + Laser + aPD‐L1 group compared to all other experimental groups. Specifically, this proportion was 3.56 times higher than that of the control group, 2.65 times that of the aPD‐L1 group, 1.49 times that of the CuS group, 1.32 times that of the Ag@CuS‐TPP@HA group, 1.19 times that of the CuS + Laser group, and 1.14 times that of the Ag@CuS‐TPP@HA + Laser group (Figure 6d). These findings substantiate our hypothesis that the combined ROS/PTT and ICB strategy could induce an immunostimulatory effect and markedly promote the maturation of tumor‐infiltrating DCs. Additionally, we observed moderate CD4^+^ T and CD8^+^ T cell infiltration in both the CuS group and Ag@CuS‐TPP@HA group. Furthermore, the populations of tumor‐infiltrating CD4^+^ T cells and CD8^+^ T cells rose by 15.8% and 12.7% respectively in the Ag@CuS‐TPP@HA+Laser and CuS+Laser groups, which suggests that the inclusion of Ag enhances the effectiveness of ROS/PTT therapy, thereby augmenting the T cell immune response. Interestingly, following treatment with Ag@CuS‐TPP@HA+Laser+aPD‐L1, the ratios of CD4^+^ T cells to CD8^+^ T cells within the mice's tumor tissue rose further by 9.9% and 8.6% respectively, compared to the Ag@CuS‐TPP@HA+Laser group (Figure 6e,f). This provides evidence that the amalgamation of ROS/PTT and ICB therapies can efficaciously stimulate the differentiation of CD4^+^ T cells and CD8^+^ T cells. Moreover, the maturation rate of DCs in spleen tissue for the Ag@CuS‐TPP@HA+Laser+aPD‐L1 group was observed to be 3.26‐fold higher than the control group (Figure 6g,h). Concurrently, CD4^+^ T cell and CD8^+^ T cell infiltration rates were significantly high, at 45.2% and 21.0% respectively (Figure 6i,j). This aligns with the data from tumor tissue infiltration rates, reinforcing the idea that the combined ROS/PTT and aPD‐L1 therapeutic strategy can enhance DC maturation. This subsequently encourages naive T cells to differentiate into CD4^+^ T and CD8^+^ T cells, a process vital for inhibiting distal tumor growth and metastasis prevention. Furthermore, post‐treatment data from the Ag@CuS‐TPP@HA+Laser+aPD‐L1 group revealed a significant reduction in the percentage of M2 tumor‐associated macrophages (TAMs) in tumor tissue to 4.3%, compared to 15.9% in the control group (Figure S9, Supporting Information). This suggests a polarization shift of M2 TAMs toward the M1 phenotype, thereby reversing the immunosuppressive TME. This polarization was further validated by the observed upregulation of IL‐12 (Figure 6k) and downregulation of IL‐10 (Figure 6l).^[^ ^14^, ^18^ ^]^ These results lend support to the hypothesis that the combination of 1064 nm laser irradiation of Ag@CuS‐TPP@HA with ICB has the potential to convert immunosuppressive “cold” tumors into immunogenic “hot” tumors. Furthermore, significantly elevated levels of IFN‐γ, TNF‐α, and IL‐6 were detected in the serum of mice in the Ag@CuS‐TPP@HA+Laser+aPD‐L1 group compared to the control (Figure 6m–o). This implies a boosted antitumor immune response in the TPP@HA+Laser+aPD‐L1 group. Overall, our results suggest that the Ag@CuS‐TPP@HA‐mediated ROS/PTT‐induced ICD, in conjunction with ICB, can stimulate an effective antitumor immune response.

The potential clinical application of Ag@CuS‐TPP@HA nanocomposites was explored by assessing their biosafety through a series of tests. Following a 16‐day treatment period, mice from each group were examined post‐mortem. Hype staining was used to analyze the major organs (heart, liver, spleen, lung, and kidney) for any possible tissue damage or inflammatory infiltration. No notable damage or inflammation was observed in any of the examined organs, as evidenced in Figure S10 (Supporting Information). The nanocomposite's biosafety was further evaluated using a hemolysis test. Even at a high concentration of 1 mg mL^−1^ of Ag@CuS‐TPP@HA, the hemolysis rate recorded was a mere 4.8% (Figure S11, Supporting Information). Additionally, the analysis of related blood biochemical indices did not show any significant alterations (Figure S12, Supporting Information). Collectively, these experimental results indicate that Ag@CuS‐TPP@HA nanocomposites exhibit excellent biocompatibility. They therefore demonstrate significant potential as a novel agent for tumor diagnosis and treatment, with considerable clinical application in precision diagnosis and efficient tumor therapy.

## 3 Conclusion

In summary, we have synthesized Ag@CuS‐TPP@HA nanoparticles by integrating Ag into near‐infrared absorbing CuS nanoparticles to form an Ag@CuS core‐shell. The surface of this construct was subsequently functionalized with TPP and HA, leading to the development of a novel nano‐diagnostic agent with dual‐targeting capabilities for cancer cells and mitochondria. This innovative approach harnesses the combined properties of ROS, photothermal reactions, and ICD antibodies to stimulate potent immunogenic tumor cell death. Consequently, this facilitates dendritic cell maturation, boosts the activation and proliferation of cytotoxic T lymphocytes, and transforms “cold” tumors with low tumor‐infiltrating lymphocyte levels into “hot” ones. This transformation stimulates T lymphocytes to more effectively recognize and destroy tumor cells, thereby enhancing the systemic antitumor immune response, which ultimately aids in eliminating primary tumors and restraining the growth of distal ones. The strategy detailed herein leverages metal element doping‐induced absorption redshift, dual targeting, and ICD antitumor therapy enhancement by CDT and PTT. This method shows promise in providing novel perspectives for the accurate diagnosis of deep‐seated tumors and the improvement of efficient antitumor immunotherapy based on ICB. Our empirical results corroborate that the proposed approach significantly ameliorates the therapeutic impact on cancer cells and metastasis. This methodology not only enables targeted NIR‐II photoacoustic imaging of tumor tissues, thereby resolving the challenge of accurate diagnosis of deep‐seated cancer tissues in vivo, but also employs CDT and PTT to augment immune checkpoints for blocking antitumor therapy, resulting in the complete eradication of primary tumors and effective inhibition of distal tumor growth. Our findings suggest that the engineered nano‐diagnostic and therapeutic agent presents substantial clinical application potential in the precise diagnosis and treatment of tumor metastasis.

## Conflict of Interest

The authors declare no conflict of interest.

## Supporting information

Supporting Information

## Data Availability

The data that support the findings of this study are available from the corresponding author upon reasonable request.

## References

[1] X. Guan , F. Polesso , C. Wang , A. Sehrawat , R. M. Hawkins , S. E. Murray , G. V. Thomas , B. Caruso , R. F. Thompson , M. A. Wood , C. Hipfinger , S. A. Hammond , J. N. Graff , Z. Xia , A. E. Moran , Nature 2022, 606, 791. 35322234 10.1038/s41586-022-04522-6 PMC10294141

[2] C. N. Spencer , J. L. McQuade , V. Gopalakrishnan , J. A. McCulloch , M. Vetizou , A. P. Cogdill , M. A. W. Khan , X. Zhang , M. G. White , C. B. Peterson , M. C. Wong , G. Morad , T. Rodgers , J. H. Badger , B. A. Helmink , M. C. Andrews , R. R. Rodrigues , A. Morgun , Y. S. Kim , J. Roszik , K. L. Hoffman , J. Zheng , Y. Zhou , Y. B. Medik , L. M. Kahn , S. Johnson , C. W. Hudgens , K. Wani , P.‐O. Gaudreau , A. L. Harris , et al., Science 2021, 374, 1632. 34941392 10.1126/science.aaz7015 PMC8970537

[3] J. R. Veatch , S. R. Riddell , Cell 2022, 185, 2848. 35931017 10.1016/j.cell.2022.07.006

[4] J. W. Smithy , M. A. Postow , Lancet 2022, 400, 1082. 36099925 10.1016/S0140-6736(22)01748-2

[5] L. Zhang , Z. Jiang , X. Yang , Y. Qian , M. Wang , S. Wu , L. Li , F. Jia , Z. Wang , Z. Hu , M. Zhao , X. Tang , G. Li , H. Shang , X. Chen , W. Wang , Adv. Mater. 2023, 35, 2207330. 10.1002/adma.202207330 36259590

[6] K. Yang , S. Qi , X. Yu , B. Bai , X. Zhang , Z. Mao , F. Huang , G. Yu , Angew. Chem., Int. Ed. 2022, 61, e202203786. 10.1002/anie.202203786 35384193

[7] H. Liang , X. Wu , G. Zhao , K. Feng , K. Ni , X. Sun , J. Am. Chem. Soc. 2021, 143, 15812. 34473493 10.1021/jacs.1c07471

[8] Y. Zhang , S. Tian , L. Huang , Y. Li , Y. Lu , H. Li , G. Chen , F. Meng , G. L. Liu , X. Yang , J. Tu , C. Sun , L. Luo , Nat. Commun. 2022, 13, 4553. 35931666 10.1038/s41467-022-32160-z PMC9356008

[9] H. Wang , Y. Chao , H. Zhao , X. Zhou , F. Zhang , Z. Zhang , Z. Li , J. Pan , J. Wang , Q. Chen , Z. Liu , ACS Nano 2022, 16, 664. 34978418 10.1021/acsnano.1c08120

[10] L. Liu , J. Chen , H. Zhang , J. Ye , C. Moore , C. Lu , Y. Fang , Y.‐X. Fu , B. Li , Nat Cancer 2022, 3, 437. 35393580 10.1038/s43018-022-00352-7 PMC9050907

[11] Y. Zhang , R. N. Sriramaneni , P. A. Clark , J. C. Jagodinsky , M. Ye , W. Jin , Y. Wang , A. Bates , C. P. Kerr , T. Le , R. Allawi , X. Wang , R. Xie , T. C. Havighurst , I. Chakravarty , A. L. Rakhmilevich , K. A. O'leary , L. A. Schuler , P. M. Sondel , K. Kim , S. Gong , Z. S. Morris , Nat. Commun. 2022, 13, 4948. 35999216 10.1038/s41467-022-32645-x PMC9399096

[12] F. Yang , Z. He , H. Duan , D. Zhang , J. Li , H. Yang , J. F. Dorsey , W. Zou , S. A. Nabavizadeh , S. J. Bagley , K. Abdullah , S. Brem , L. Zhang , X. Xu , K. T. Byrne , R. H. Vonderheide , Y. Gong , Y. Fan , Nat. Commun. 2021,12, 3424. 34103524 10.1038/s41467-021-23832-3 PMC8187342

[13] G. Yu , F. Dong , W. Ge , L. Sun , L. Zhang , L. Yuan , N. Li , H. Dai , L. Shi , Y. Wang , Nano Today 2022, 44, 101498.

[14] D. Wang , M. Zhang , Y. Zhang , G. Qiu , J. Chen , X. Zhu , C. Kong , X. Lu , X. Liang , L. Duan , C. Fang , J. Liu , K. Zhang , T. Luo , Adv. Sci. 2022, 9, 2203106. 10.1002/advs.202203106 PMC9661857 36156442

[15] Y. Li , X. Jiang , T. Luo , J. Xia , M. J. Lee , R. R. Weichselbaum , W. Lin , Biomaterials 2022, 290, 121831. 36240687 10.1016/j.biomaterials.2022.121831

[16] W. Jiang , W. Dong , M. Li , Z. Guo , Q. Wang , Y. Liu , Y. Bi , H. Zhou , Y. Wang , ACS Nano 2022, 16, 3881. 35238549 10.1021/acsnano.1c09048

[17] P. Pan , X. Dong , Y. Chen , J.‐J. Ye , Y.‐X. Sun , X.‐Z. Zhang , Biomaterials 2022, 289, 121810. 36152517 10.1016/j.biomaterials.2022.121810

[18] X. Li , J. Pan , Y. Li , F. Xu , J. Hou , G. Yang , S. Zhou , ACS Nano 2022, 16, 5778. 35324153 10.1021/acsnano.1c10892

[19] J. Tan , B. Ding , P. Zheng , H. Chen , P. Ma , J. Lin , Small 2022, 18, 2202462. 10.1002/smll.202202462 35896867

[20] K. Wang , J. Chen , L. Lin , N. Yan , W. Yang , K. Cai , H. Tian , X. Chen , Nano Today 2022, 46, 101579.

[21] Z. Li , Z. Chu , J. Yang , H. Qian , J. Xu , B. Chen , T. Tian , H. Chen , Y. Xu , F. Wang , ACS Nano 2022, 16, 15471. 35981098 10.1021/acsnano.2c08013

[22] Z. Gao , S. Jia , H. Ou , Y. Hong , K. Shan , X. Kong , Z. Wang , G. Feng , D. Ding , Angew. Chem., Int. Ed. 2022, 61, e202209793. 10.1002/anie.202209793 35916871

[23] Y. Zhao , Z. Chen , Q. Li , X. Cao , Q. Huang , L. Shi , Y. Liu , Adv. Funct. Mater. 2022, 32, 2209711.

[24] J. Qi , S. Jia , X. Kang , X. Wu , Y. Hong , K. Shan , X. Kong , Z. Wang , D. Ding , Adv. Mater. 2022, 34, 2203309. 10.1002/adma.202203309 35704513

[25] Z. Fan , Y. Wang , L. Li , F. Zeng , Q. Shang , Y. Liao , C. Liang , L. Nie , ACS Nano 2022, 16, 16177. 36136614 10.1021/acsnano.2c04983

[26] Y. Yang , J. Ge , G. Li , H. Lei , L. Chen , Y. Gong , X. Zhong , L. Wang , Y. Dai , W. Tang , J. Zou , Y. Cheng , Z. Liu , L. Cheng , Nano Today 2022, 46, 101585.

[27] B. Ding , P. Zheng , F. Jiang , Y. Zhao , M. Wang , M. Chang , P. '. Ma , J. Lin , Angew. Chem., Int. Ed. 2020, 59, 16381. 10.1002/anie.202005111 32484598

[28] L. Zhou , P. Zhang , H. Wang , D. Wang , Y. Li , Acc. Chem. Res. 2020, 53, 1761. 32819102 10.1021/acs.accounts.0c00254

[29] Z. Wang , L. Yu , Y. Wang , C. Wang , Q. Mu , X. Liu , M. Yu , K.‐N. Wang , G. Yao , Z. Yu , Adv. Sci. 2022, 9, 2104793. 10.1002/advs.202104793 PMC8922098 35064653

[30] Z. Li , X. Lai , S. Fu , L. Ren , H. Cai , H. Zhang , Z. Gu , X. Ma , K. Luo , Adv. Sci. 2022, 9, 2201734. 10.1002/advs.202201734 PMC9353475 35652198

[31] B. Tian , C. Wang , Y. Du , S. Dong , L. Feng , B. Liu , S. Liu , Small 2022,18, 2200786. 10.1002/smll.202200786 35661402

[32] G.‐F. Luo , J.‐L. Liang , D.‐W. Zheng , P. Ji , J.‐W. Wang , W.‐H. Chen , X.‐Z. Zhang , Adv. Funct. Mater. 2022, 32, 2205550.

[33] H. Jiang , H. Fu , Y. Guo , P. Hu , J. Shi , Biomaterials 2022, 289, 121799. 36152515 10.1016/j.biomaterials.2022.121799

[34] S. Fulda , L. Galluzzi , G. Kroemer , Nat. Rev. Drug Discovery 2010, 9, 447. 20467424 10.1038/nrd3137

[35] D. C. Wallace , Nat. Rev. Cancer 2012, 12, 685. 23001348 10.1038/nrc3365 PMC4371788

[36] A. P. Jathoul , J. Laufer , O. Ogunlade , B. Treeby , B. Cox , E. Zhang , P. Johnson , A. R. Pizzey , B. Philip , T. Marafioti , M. F. Lythgoe , R. B. Pedley , M. A. Pule , P. Beard , Nat. Photonics 2015, 9, 239.

[37] A. Taruttis , V. Ntziachristos , Nat. Photonics 2015, 9, 219.

[38] C. Xu , Y. Jiang , Y. Han , K. Pu , R. Zhang , Adv. Mater. 2021, 33, 2008061. 10.1002/adma.202008061 33634897

[39] X. Wang , X. Wang , Q. Yue , H. Xu , X. Zhong , L. Sun , G. Li , Y. Gong , N. Yang , Z. Wang , Z. Liu , L. Cheng , Nano Today 2021, 39, 101170.

[40] W. Zeng , X. Wu , T. Chen , S. Sun , Z. Shi , J. Liu , X. Ji , X. Zeng , J. Guan , L. Mei , M. Wu , Adv. Funct. Mater. 2021, 31, 2008362.

[41] C. Ou , W. Na , W. Ge , H. Huang , F. Gao , L. Zhong , Y. Zhao , X. Dong , Angew. Chem 2021, 133, 8238. 10.1002/anie.202014852 33432650

[42] Y. Sun , P. Zhang , Y. Li , Y. Hou , C. Yin , Z. Wang , Z. Liao , X. Fu , M. Li , C. Fan , D. Sun , L. Cheng , ACS Nano 2022, 16, 18667. 36264835 10.1021/acsnano.2c07311

[43] T. Chen , Z. Chen , Q. Zhou , H. Ding , P. Gong , J. Wang , H. Cai , R. Ao , M. Yu , J. Song , L. Lin , H. Yang , Adv. Funct. Mater. 2022, 32, 2208720.

[44] J. M. Luther , P. K. Jain , T. Ewers , A. P. Alivisatos , Nat. Mater. 2011, 10, 361. 21478881 10.1038/nmat3004

[45] F. Li , Y. Liu , Y. Dong , Y. Chu , N. Song , D. Yang , J. Am. Chem. Soc. 2022, 144, 4667. 35254064 10.1021/jacs.2c00823

