# Supplementary material for: A Silver‐Induced Absorption Red‐Shifted Dual‐Targeted Nanodiagnosis‐Treatment Agent for NIR‐II Photoacoustic Imaging‐Guided Photothermal and ROS Simultaneously Enhanced Immune Checkpoint Blockade Antitumor Therapy
Source: Adv Sci (Weinh). 2023 Dec 31;11(11):2306375. doi: 10.1002/advs.202306375 (PMC10953570; doi:10.1002/advs.202306375)
Supplement: Supplementary file 1 — Supporting Information [file ADVS-11-2306375-s001.pdf]

ADVANCED SCIENCE
Open Access

Supporting Information

for *Adv. Sci.*, DOI 10.1002/advs.202306375

A Silver-Induced Absorption Red-Shifted Dual-Targeted Nanodiagnosis-Treatment Agent for NIR-II Photoacoustic Imaging-Guided Photothermal and ROS Simultaneously Enhanced Immune Checkpoint Blockade Antitumor Therapy

Yulong Bai, Jing Hua, Jingjin Zhao, Shulong Wang, Mengjiao Huang, Yang Wang, Yanni Luo, Shulin Zhao* and Hong Liang**

Supporting Information

A Silver-Induced Absorption Red-Shifted Dual-Targeted Nanodiagnosis-Treatment Agent for NIR-II Photoacoustic Imaging-Guided Photothermal and ROS Simultaneously Enhanced Immune Checkpoint Blockade Antitumor Therapy

Yulong Bai,[1,2,+] Jing Hua,[1,+] Jingjin Zhao,[1,] Shulong Wang,[1] Mengjiao Huang,[1] Yang Wang,[1] Yanni Luo,[1] Shulin Zhao,[1,]* and Hong Liang[1,]**

[1] State Key Laboratory for the Chemistry and Molecular Engineering of Medicinal Resources, School of Chemistry and Pharmaceutical Science, Guangxi Normal University, Guilin, 541004, China.

[2] School of Medicine, Shanghai Research Institute for Intelligent Autonomous Systems, Tongji University, Shanghai, 200092, China.

Experimental Section

Materials and Reagents. Copper chloride dihydrate, sodium citrate, silver nitrate, and sodium sulfide hexahydrate were sourced from Shanghai Aladdin Biochemical Technology Co., Ltd. Enzymes including collagenase IV, deoxyribonuclease I (DNAse I), and hyaluronidase (HAase) were procured from Beijing Solebao Technology Co., Ltd. Elisa kits for mouse calreticulin (CRT), high mobility group protein B1 (HMGB1), and heat shock protein-90 (HSP-90) were obtained from Shanghai Enzyme Union Biotechnology Co., Ltd. (Mlbio). The enhanced ATP

detection kit was acquired from Shanghai Biyuntian Biotechnology Co., Ltd. (Beyotime). Cytokines IL-6, IL-10, IL-12, TNF- α, and IFN- γ were secured from Elabscience. The following antibodies were purchased from Biolegend: Anti-CDF4/80-PerCP-Cy5.5, Anti-CD206-Alexa Fluor 647, Anti-CD11c-PE-Cy7, Anti-CD80-Alexa Fluor 488, Anti-CD86-PE, Anti-CD45-FITC, Anti-CD3-Alexa Fluor 488, Anti-CD4-PerCP-Cy5.5, and Anti-CD8-Alexa Fluor 647. Two cell lines, 4T1 (mouse breast cancer cells) and HL-7702 (human normal liver cells), were obtained from the Cell Bank of the Typical Culture Preservation Committee of the Chinese Academy of Sciences / Cell Resource Center of Shanghai Academy of Life Sciences. BALB/c mice were supplied by Hunan Shrek Jingda Experimental Animal Co., Ltd. All other chemical reagents employed in the experiments were of pure analytical grade, and the experimental water used exhibited a resistivity of 18.2 MΩ·cm.

Synthesis of Ag@CuS. In a 200 mL round-bottom flask containing 80 mL of deionized water, 40 mg of $CuCl_2 \cdot 2H_2O$, 20 mg of $AgNO_3$, and 45 mg of sodium citrate were combined and uniformly mixed. This solution was then heated to 40° C while being continuously stirred. Subsequently, $Na_2S \cdot 9H_2O$ (20 mL, 1 mg/mL) was added to the flask, with the stirring and heating process continued until the mixture reached 95°C, at which point the conditions were maintained for one hour. The resultant solution was dialyzed and filtered in deionized water for a period of 6-8

hours, and finally stored in a refrigerator at 4°C.

Synthesis of Ag@CuS-TPP@HA. A solution of synthetic Ag@CuS (0.4 mg/mL, 10 mL) and a polyallylamine hydrochloride solution (PAH, 5 mM, 5 mL) were mixed in a 50 mL round-bottom flask and stirred at 37°C for two hours. To this mixture, 2 mL of EDC (0.1 M) and 2 mL of NHS (0.1 M) were added and further stirred at 37°C for two hours. TPP (5 mL, 5 mM) was then added, and the solution was stirred for another two hours at 37°C, followed by a centrifugation process at 5000 rpm for 10 minutes to obtain a light brown Ag@CuS-TPP aqueous solution. The final step involved the addition of HA (3 mL, 3 mg/mL), stirring for two more hours,

and obtaining the dark black Ag@CuS-TPP@HA solution, which was stored in a refrigerator at 4°C.

Detection of Cell ROS/JC-1. Cells were cultured in a confocal culture dish at a density of 1×10^5 cells per plate for 24 hours. They were then incubated with fresh medium containing PBS, CuS, and Ag@CuS-TPP@HA (400 μg/mL) for four hours. Six experimental groups were established: Control, PBS+Laser, CuS, Ag@CuS-TPP@HA, CuS+Laser, and Ag@CuS-TPP@HA+Laser. In the groups with laser treatment, cells were exposed to a 1064 nm laser (1.0 W/cm^2) for five minutes. Cellular ROS levels in each group were subsequently detected via DCFH-DA staining, while mitochondrial membrane potential alterations were observed using JC-1 staining. Finally, fluorescence imaging of each Petri dish was conducted with a two-photon laser confocal microscope, and the cell suspensions were collected for flow cytometry analysis using a Thermo Fisher Scientific Attune NxT cytometer (USA).

Detection of Immunogenic Cell Death Induced in Vitro. Cells were cultured in a six-well plate at a density of 1×10^5 cells per well for 24 hours, followed by incubation in fresh medium supplemented with PBS, CuS, or Ag@CuS-TPP@HA (400 μg/mL) for an additional four hours. The cell cultures were divided into six groups: Control, PBS+Laser, CuS, Ag@CuS-TPP@HA, CuS+Laser, and Ag@CuS-TPP@HA+Laser. The groups denoted with "Laser" were subjected to

irradiation with a 1064 nm laser (1.0 W/cm^2) for five minutes. After an additional four-hour incubation, the supernatants were collected. Levels of CRT, HMGB1, HSP-90, and ATP—markers related to immunogenic cell death—were quantified using the respective CRT, HMGB1, and HSP-90 enzyme-linked immunosorbent assay kits (Shanghai Enzyme-Linked Biotechnology Co., Ltd.) as well as the enhanced ATP detection kit (Shanghai Biyuntian Biotechnology Co., Ltd.).

Animals experiments. BALB/c mice, aged between 6 and 8 weeks, were procured from Hunan SJA Laboratory Animal Co., Ltd. (Changsha, China). All animal handling processes adhered to the Animal Ethics Committee guidelines of

Guangxi Normal University (Approval No. 202203-003). The mice were maintained under specific-pathogen-free conditions with unrestricted access to standard food and water. All animal studies complied with the guidelines established by the National Regulation of China for Care and Use of Laboratory Animals.

PA Imaging. The PA performance of Ag@CuS-TPP@HA was assessed using the MSOT Invision 256-TF multispectral PA imaging system (iThera Medical GmbH, Munich, Germany). PA imaging was conducted on the PBS group, the CuS group, the Ag@CuS group, and the Ag@CuS-TPP@HA group (1 mg/mL), with the corresponding PA signal intensity being monitored. For in vivo PA imaging, Ag@CuS-TPP@HA was administered to mice via tail vein injection, followed by monitoring of the tumor site in tumor-bearing mice via in situ NIR-II (1064 nm) PA imaging over a 12-hour period.

Photothermal Imaging in Vivo. Each group of agents was administered to mice via tail vein injection. Eight hours post-injection, a 1064 nm laser (1.0 W/cm^2) was used to irradiate the tumor sites for five minutes. The temperature changes within the tumors were recorded in real time using an infrared thermal imager (OptrisPI-160), and thermal imaging analysis was performed using the imager’s proprietary software.

ROS/Photothermal Enhanced Immune Checkpoint Blocking Antitumor Therapy in Tumor-Bearing Mice. The procedure involved injecting 1×10^5 4T1 cells subcutaneously into the left side of the dorsal region of female BALB/c mice, which

was designated as the distal tumor model. Conversely, 1×10^6 4T1 cells were similarly administered subcutaneously into the right side, creating the primary tumor model. Following the growth of the primary tumor to approximately 100 mm^3, the 4T1 tumor-bearing mice were categorized into seven groups, each containing five subjects: the control group (Group 1), the anti-PD-L1 group (Group 2), the CuS group (Group 3), the Ag@CuS-TPP@HA group (Group 4), the CuS plus Laser group (Group 5), the Ag@CuS-TPP@HA plus Laser group (Group 6), and the Ag@CuS-TPP@HA plus Laser and anti-PD-L1 group (Group 7). Each group received an intravenous tail injection of their corresponding therapeutic agents (100 μL at 1 mg/mL) and, after

eight hours, the primary tumor sites of mice from Groups 5, 6, and 7 were exposed to 1064 nm laser irradiation (1.0 W/cm^2) every other day. In addition, on Days 1, 3, and 5 of the experiment, mice from Groups 2 and 7 received an intraperitoneal injection of anti-PD-L1, with a dosage of approximately 30 μL (1 mg/mL) per mouse. Throughout the 16-day treatment period, the body weight and primary and distal tumor volume changes for each mouse were measured and recorded bi-daily. Tumor volume was ascertained using a Vernier caliper and calculated following the formula: tumor volume = (tumor length) × (tumor width) ^{2}/ 2.

In Vitro Assays for Different Immune Cells. BALB/c female mice were subcutaneously injected with 1×10^5 4T1 cells, serving as a distal tumor model, into the left side of the back. After eight days, the tumor-bearing mice were euthanized, and both the left tumor (distal tumor) tissues and spleen tissues were harvested from each group. The tissues were then subjected to digestion with a solution containing 0.2% collagenase IV, 0.01% hyaluronidase, and 0.002% deoxyribonuclease I at 37 °C for a duration of 30 minutes. Following digestion, the tissues were filtered through a 40 µm cell filter membrane and rinsed with phosphate-buffered saline. The resultant cell suspensions were stained with Anti-CD45-FITC, Anti-CD3-Alexa Fluor 488, anti-PerCP-Cy5.5 CD4-PerCP-Cy5.5, and Anti-CD8-Alexa Fluor 647 antibodies. Flow cytometry was then employed to determine the populations of $CD4^+$ and $CD8^+$ T cells within the tumor and spleen tissues, as well as to assess the maturation rate of

DCs. Lastly, the phenotypic alterations in macrophages within the tumor tissues were observed using flow cytometry, following staining with Anti-CDF4/80-PerCP-Cy5.5 and Anti-CD206-Alexa Fluor 647 antibodies.

Study of Biological Distribution and Metabolism. Mice were intravenously administered Ag@CuS-TPP@HA (1 mg/mL, 200 μL), after which they were euthanized at time points of 2, 12, and 24 hours post-injection. The major organs, namely the heart, liver, spleen, lungs, and kidneys, along with tumor tissues, were harvested. All harvested biological tissue samples were subsequently digested and analyzed via inductively coupled plasma optical emission spectroscopy (ICP-OES),

thereby determining the concentration (μg/g) of Cu and Ag in each tissue sample.

Data Analysis. All experimental data are expressed as the mean ± standard deviation (SD). Comparisons of statistical differences between two groups were conducted using Student's *t*-test, while single-factor analysis of variance (ANOVA), performed using GraphPad Prism 8.0 software, was utilized for multi-group comparisons. Statistically significant differences were denoted by $^*P < 0.05$, $^{**}P < 0.01$ and $^{***}P < 0.001$ respectively.

Supporting Diagram

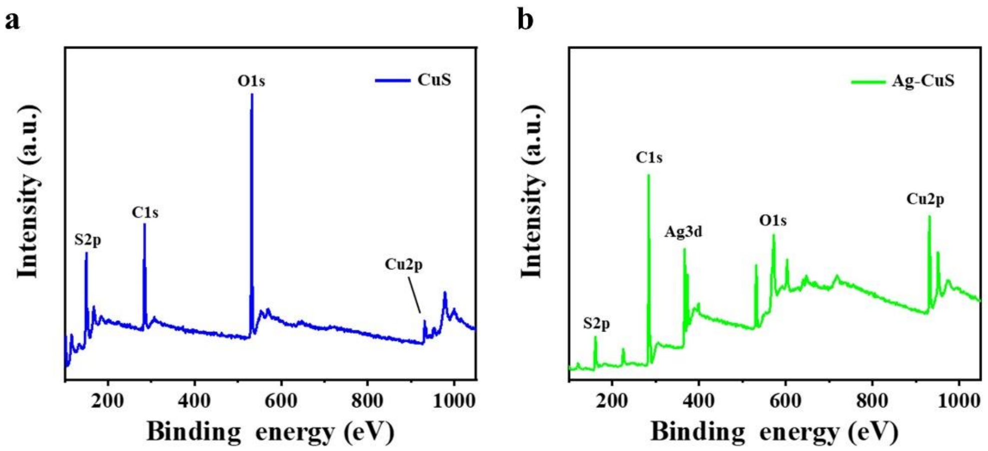

Figure S1. XPS maps of CuS (a) and Ag@CuS (b).

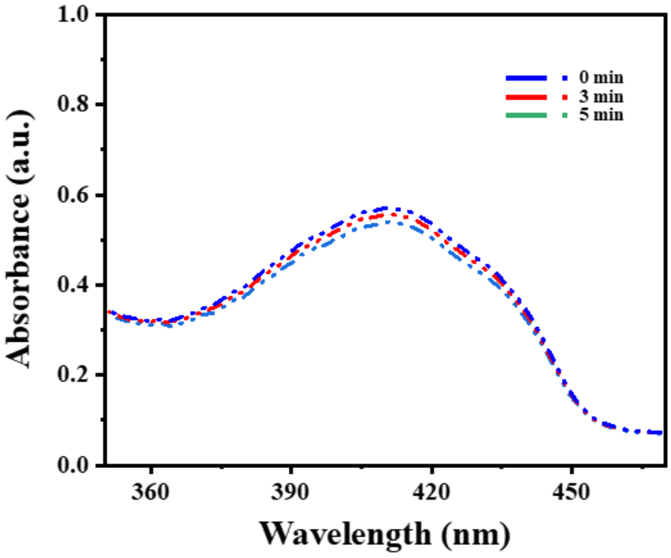

Figure S2. The UV-visible absorption curve of DPBF solution (0.1mM) in the presence of Ag@CuS-TPP@HA in phosphate buffer, irradiated with 1064 nm (1.0 W/cm^2) laser.

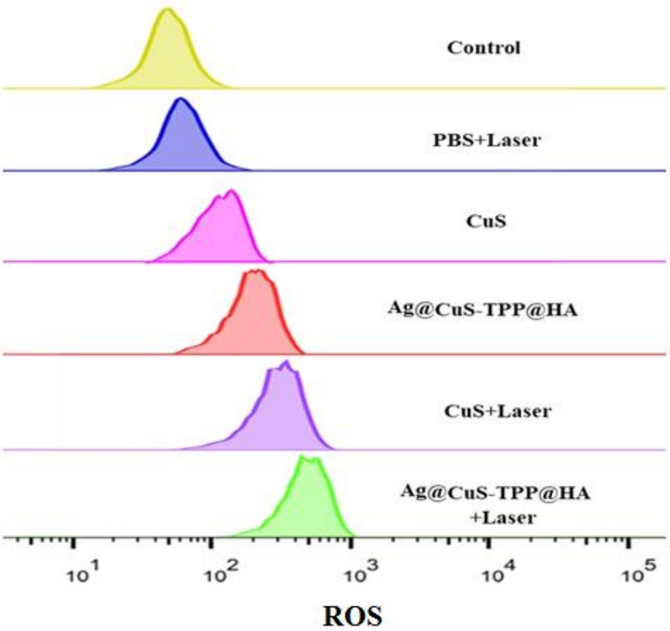

Figure S3. Analysis of intracellular reactive oxygen species production by flow cytometry.

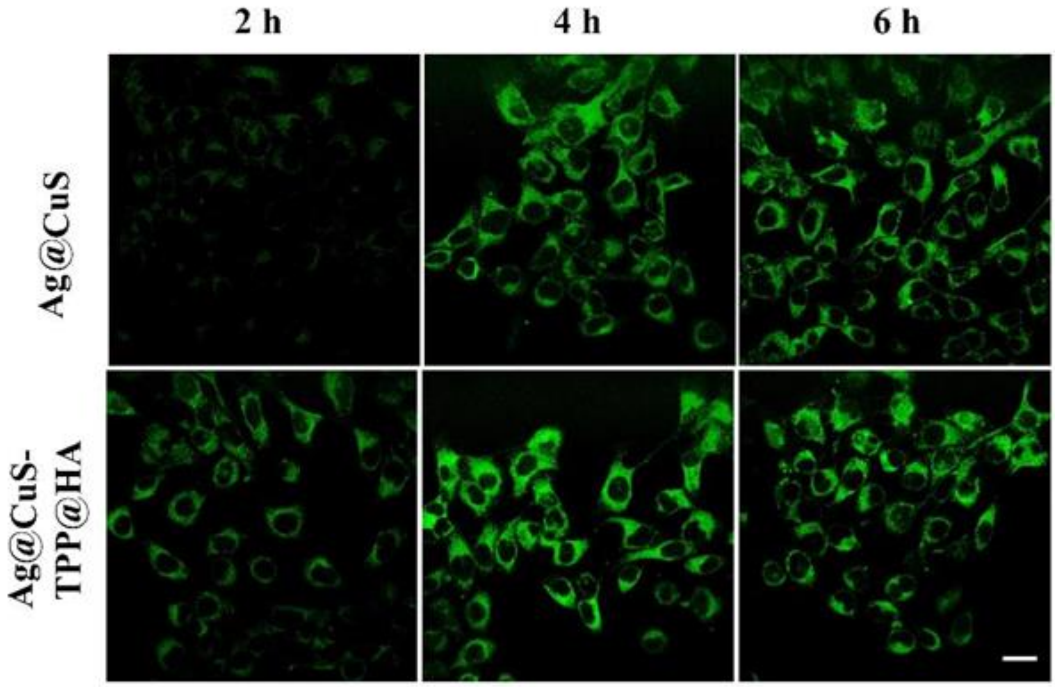

Figure S4. Confocal fluorescence imaging of cells incubated with Ag@CuS and Ag@CuS-TPP@HA at different time points : λ_{ex}=405 nm, λ_{em}=415-650 nm, Scale bar: 20 µm.

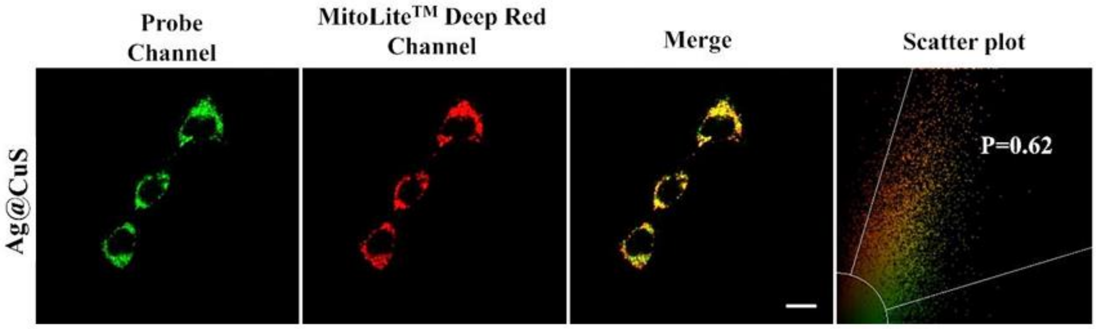

Figure S5. Co-located fluorescence imaging of Ag@CuS in subcellular organelles. Green channel: λ_{ex} =405 nm, λ_{em} =415-650 nm; red channel: λ_{ex} =633 nm, λ_{em} =643-800 nm. Scale bar: 10 µm.

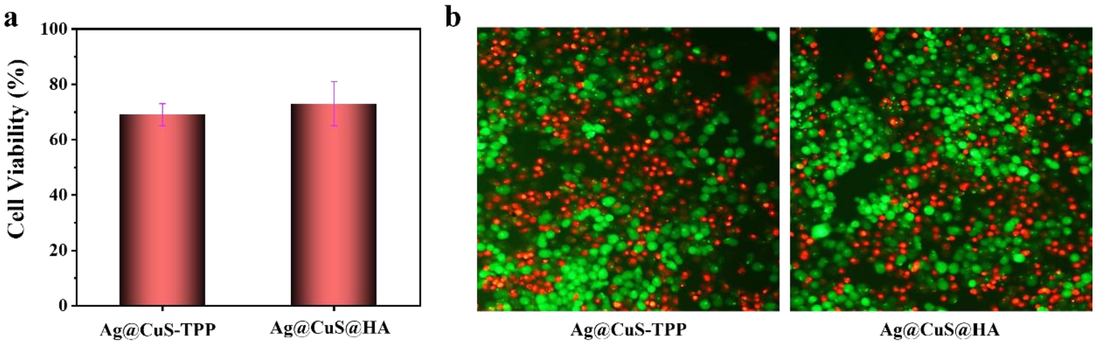

Figure S6. (a) The survival rate of 4T1 cells incubated, respectively with Au@CuS-TPP and Ag@CuS@HA solution for 24 h, and (b) the staining map of living / dead cells.

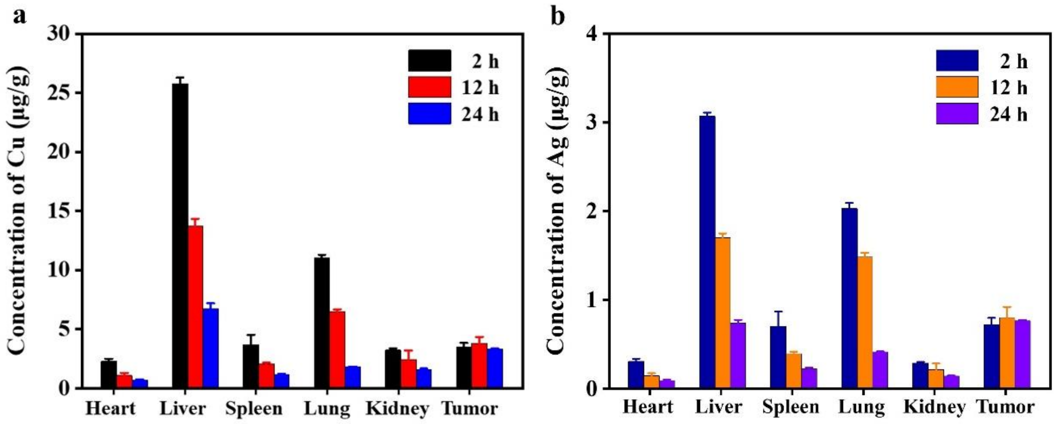

Figure S7. Tissue distribution of Cu (a) and Ag (b) in main organs after tail vein injection of Ag@CuS-TPP@HA solution at different time points.

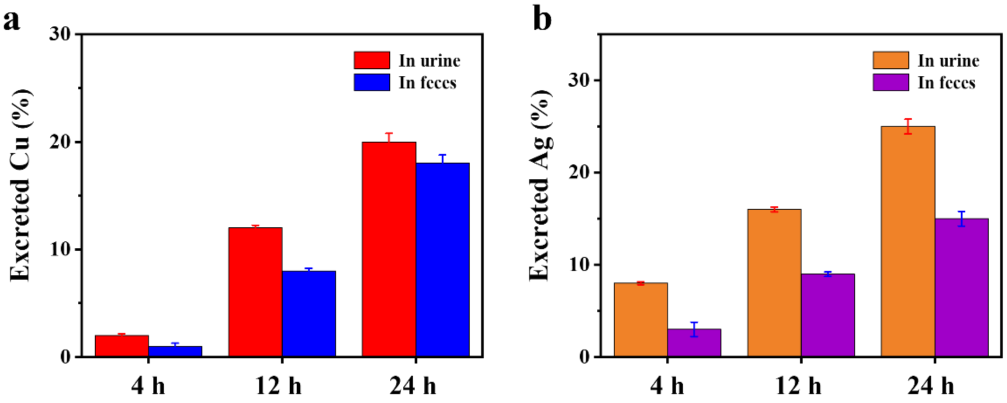

Figure S8. The contents of Cu (a) and Ag (b) in feces and urine of tumor-bearing mice at different time points after intravenous injection of Ag@CuS-TPP@HA.

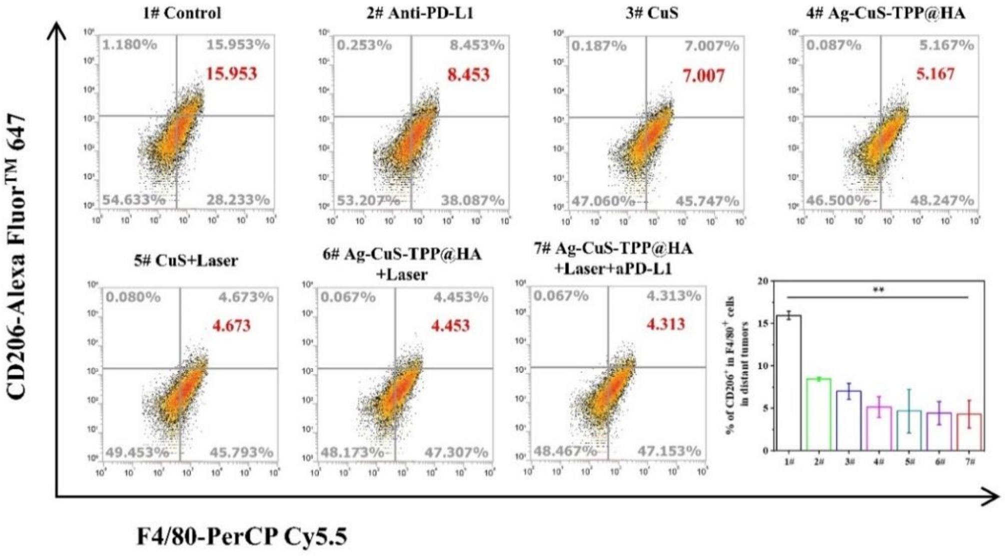

Figure S9. The tumor tissue cell suspensions of tumor-bearing mice were collected after 8 days of treatment with different therapeutic agents, and the results of M2 macrophages were determined by flow cytometry after staining with F4/80 and CD206.

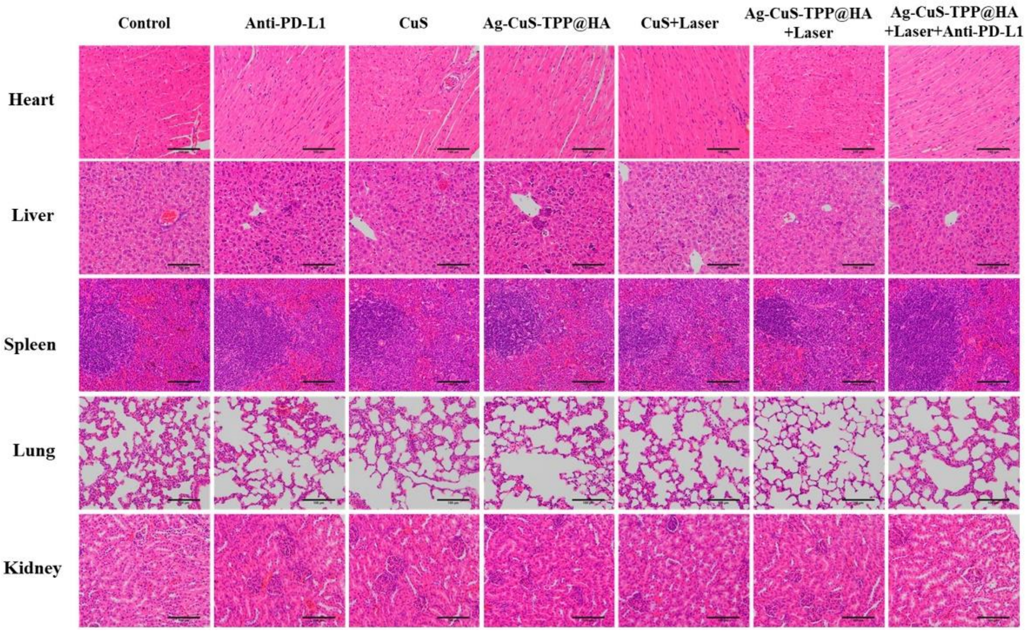

Figure S10. Tissue section analysis of main organs of mice in different treatment groups after 16 days of treatment. Scale bar: 100 μm.

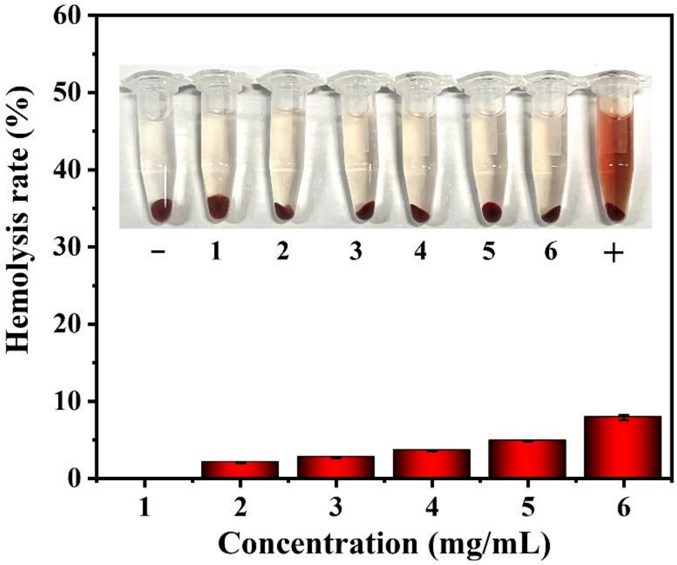

Figure S11. Blood compatibility analysis of Ag@CuS-TPP@HA nanocomposites. The error bar represents the standard deviation of three independent measurements.

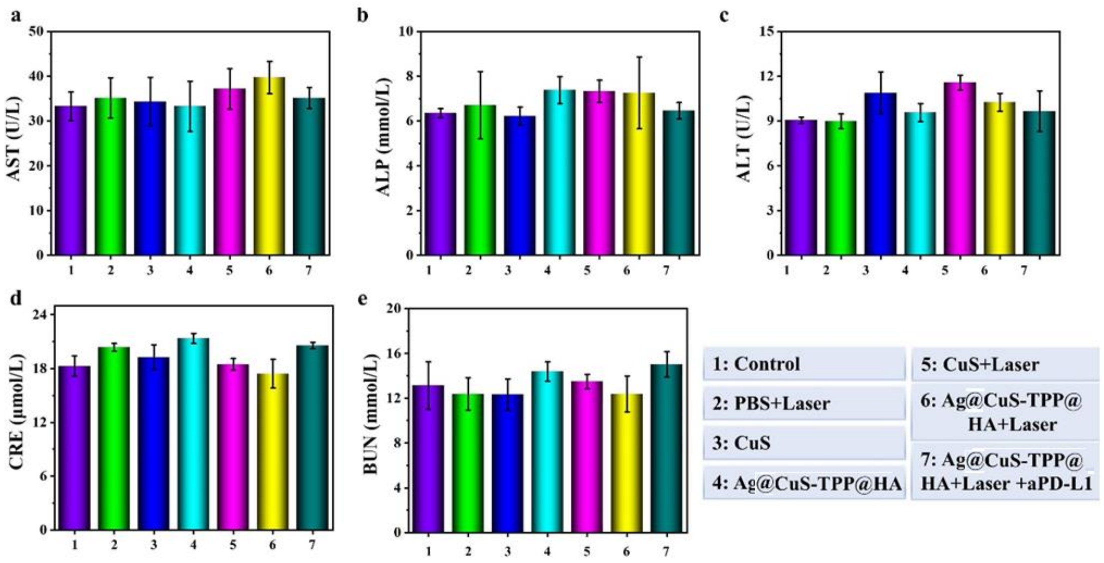

Figure S12. The related blood biochemical indexes of tumor-bearing mice in different treatment groups were tested 14 days after treatment. The error bar represents the standard deviation of three independent measurements.
